# Healthcare use and its variation in people with fibromyalgia: a systematic review protocol

**DOI:** 10.1017/S1463423625000362

**Published:** 2025-05-07

**Authors:** Ailish Katherine Byrne, Helen Twohig, Sara Muller, Ian C. Scott

**Affiliations:** 1 Primary Care Centre Versus Arthritis, School of Medicine, Keele University, Keele, UK; 2 Haywood Academic Rheumatology Centre, Haywood Hospital, Midlands Partnership University NHS Foundation Trust, High Lane, Burslem, Staffordshire, UK

**Keywords:** fibromyalgia, systematic review, meta-analysis, healthcare use, electronic health record

## Abstract

**Aim:**

A crucial step towards improving the care of people with fibromyalgia is understanding current practice. Our systematic review aims to address this by synthesising the global evidence around healthcare use in people with fibromyalgia, including its variation across groups of people, geographical locations, and over time.

**Background:**

Fibromyalgia is a chronic condition characterized by widespread pain alongside a broad range of non-pain symptoms. Its substantial impact on peoples’ lives and high prevalence mean that ensuring people with fibromyalgia receive evidence-based and appropriate care is a clinical and research priority. Whilst guidelines recommend that people with fibromyalgia receive a prompt diagnosis, care that focuses on non-pharmacological interventions, and in many countries should be predominantly managed in the community, existing evidence indicates they often wait many years for a diagnosis, commonly receive long-term opioid medicines, and see multiple hospital specialists.

**Methods:**

Relevant databases will be searched, with 25% of screening, data extraction, and quality appraisal conducted by two reviewers. Eligible studies will have evaluated healthcare use in adults with fibromyalgia using data obtained from electronic health record, registry, or insurance databases (providing generalizable findings in large, representative datasets). Data will be synthesized using meta-analysis and/or synthesis without meta-analysis where possible.

**Results:**

By providing an in-depth analysis of healthcare use and its variation in people with fibromyalgia, the results from this systematic review could be used to benchmark practice, inform targeted management strategies to those with the highest levels of healthcare use (and therefore care need), and provide insight into whether certain countries require specific guideline/policy changes.

## Background

Fibromyalgia is a chronic condition, characterized by widespread pain and tenderness alongside a broad range of non-pain symptoms, including fatigue, cognitive disturbance, dizziness, and breathlessness (Berwick, Barker & Goebel, 2022). Its high estimated prevalence (ranging from 1.7% to 5.4%, depending on the classification criteria applied) (Jones et al., 2015) and detrimental impact on patients’ quality of life, ability to work and function (Arnold, Gebke & Choy, 2016) mean that ensuring people with fibromyalgia receive evidence-based and appropriate healthcare is a clinical and research priority (Macfarlane et al., 2017; National Institute for Health and Care Excellence, 2021).

Despite this, there is substantial evidence that people with fibromyalgia have suboptimal care, leaving both patients and clinicians dissatisfied (Gharibpoor et al., 2021; Byrne et al., 2023). This can be considered from three broad aspects. First, although recent years have seen a simplification in the approach to diagnosing fibromyalgia (now made based on the presence of self-reported widespread pain and severe somatic symptoms for ≥3 months (RCP, 2022)), and recommendations in many countries (such as the UK, Canada, and Australia) that fibromyalgia should predominantly be managed in community and primary care services (Endresen, 2007; Fitzcharles et al., 2013; Kay et al., 2021; Fibromyalgia Australia, 2023), people with fibromyalgia often wait years to receive a diagnosis (Choy et al., 2010), seeing multiple specialists and undergoing numerous investigations in the intervening time (Arnold, Gebke & Choy, 2016). This is at odds with guidelines, which advocate a prompt diagnosis to ensure optimal management (Macfarlane et al., 2017). Second, whilst guidelines now recommend a focus on non-pharmacological care involving exercise, psychological therapies, and acupuncture (Macfarlane et al., 2017; National Institute for Health and Care Excellence, 2021), people with fibromyalgia are often prescribed analgesics (particularly long-term opioids and gabapentinoids) in-spite of weak supportive evidence for efficacy, high cumulative analgesic costs (Painter et al., 2015), and many potential harms (Valladales-Restrepo et al., 2022). Third, even after receiving a diagnosis, people with fibromyalgia continue to undergo many investigations and specialist reviews owing to the broad range of symptoms they can experience (Arnold, Gebke & Choy, 2016).

A crucial step towards optimising the care that people with fibromyalgia receive is to understand current practice. Whilst a recent systematic review (D’Onghia et al., 2022) has synthesized existing research around economic costs in people with fibromyalgia (estimating it to be six times higher than the general population), that review’s focus was on the components and predictors of these costs (spanning medical, non-medical, and indirect costs), not an exploration of the types and patterns of healthcare use. At present, no similar in-depth review exists for healthcare use in people with fibromyalgia.

As care for people with fibromyalgia in many countries is increasingly delivered in the community, profiling the nuances of healthcare use is essential if medicine is to move towards tailored, person-centred, and evidence-based care for fibromyalgia that is feasible within primary care’s resource-limited setting (McConnell, Heron & Hart, 2023). This is also relevant to countries in which fibromyalgia is predominantly managed in secondary care, in which resources are also finite. Our systematic review will address this evidence gap by delivering the following three inter-related objectives in people with fibromyalgia: to (1) describe levels of different aspects of healthcare use (benchmarking practice); (2) examine whether there are certain patient groups with very high levels of healthcare use (informing targeted care strategies to those with the highest care need); and (3) evaluate how this varies across geographical regions and over time (giving insight into whether certain countries may require specific guideline/policy changes, and whether management approaches are changing over time).

## METHODS

### Registration

This protocol was registered with the International Prospective Register of Systematic Reviews (PROSPERO, CRD 531899). No similar protocol exists at the time of writing. This manuscript was prepared adhering to the Preferred Reporting Items for Systematic Review and Meta-Analysis Protocols (PRISMA-P, Supplementary Material 1) checklist (Moher et al., 2015). Any protocol amendments will be reported within the supplementary material of the completed review and the PROSPERO record.

### Search Strategy

A pilot search strategy was generated for the database MEDLINE by the corresponding author (AB). A review of the strategy’s sensitivity and specificity was completed via informal title screening of 500 articles. It was subsequently refined with clinical academics in rheumatology (ICS) and general practice (HT), alongside an academic in epidemiology and statistics (SM) and an information specialist (acknowledgements). Once the final comprehensive search strategy was agreed for MEDLINE it was then tailored for the remaining databases: Web of Science, EMBASE and CINHAL Plus (Supplementary Material 2). Reference lists of studies included in full-text review and any relevant systematic reviews emerging from screening will be manually searched.

### Inclusion Criteria

Population, exposure, comparator, and outcome (PECO, McKenzie et al., 2023) criteria (Table 1) supported inclusion criteria development. Studies will be included that: a) include adults aged ≥18 years with a diagnosis of fibromyalgia; b) are full-text publications exploring healthcare use of any type and in any setting; c) are large, representative observational studies in routinely collected electronic health record (EHR), registry, or insurance databases; and d) were published from 1990-2024 (with fibromyalgia existing as a clearly defined clinical entity under this term since the 1990 American College of Rheumatology classification criteria publication) (Wolfe et al., 1990).

**Table 1.** Systematic Review Population, Exposure, Comparator, and Outcome (PECO) Criteria

PECO criterion	Details
*Population of interest*	Adults (≥18 years of age) with a diagnosis of fibromyalgia.
*Exposures of interest*	(a.) Geographical location and healthcare models; (b.) calendar time; (c.) patient characteristics (e.g., age, gender, ethnicity, socio-demographics, comorbidities).
*Comparisons or control groups*	No control group is being specified or required, but individual studies may include a control or comparator group.
*Outcomes of interest*	Healthcare resource use, including (but not limited to): (a.) primary care consultations; (b.) outpatient visits; (c.) analgesic prescriptions; (d.) overall prescriptions; (e.) hospitalisations; (f.) investigations; (g.) healthcare costs.
*Setting*	Any health or care setting in any country.
*Study designs*	Large, representative observational studies in routinely collected electronic health record, registry, or insurance databases.

### Exclusion Criteria

Studies will be excluded that: a) do not report healthcare use in people with fibromyalgia (or contain only aggregate data from people with fibromyalgia mixed with other populations); b) use experimental (e.g., trial) study designs; c) are observational studies not conducted in, or linked to, routinely collected EHR, registry, or insurance databases; d) are abstracts, protocols, systematic reviews, meta-analyses or editorials; e) use qualitative methods; f) are foreign language articles for which a translation cannot be obtained (via translation software/native speakers); g) assess healthcare use in people with fibromyalgia explicitly in relation to a comorbid condition (e.g., in people with fibromyalgia receiving hip replacement surgery).

### Study Screening and Selection

Search results will be imported into the reference management software Rayyan (Ouzzani, Hammady, Fedorowicz & Elmagarmid, 2016) and duplicates removed. Title, abstract and full-text screening will be conducted. Rayyan provides the capacity to add labels to each record, allowing decisions to be transparently recorded throughout screening. For each stage of screening, two independent reviewers will screen 25% of data, with disagreements resolved by discussion, deferring to a third reviewer where consensus cannot be achieved. The remaining 75% of studies will be assessed by one reviewer. In instances where there is disagreement on >10% of articles, an inter-rater reliability assessment, conducted using Cohen’s Kappa, will be reported.

### Primary and Secondary Outcomes

The primary outcome is healthcare use. We anticipate this will be divisible into counts/prevalence/rates of: a) primary care consultations; (b) outpatient visits; (c) drug prescriptions (including analgesics); (d) hospitalizations; and (f) investigations. The secondary outcome is healthcare costs.

### Data Extraction

Two independent reviewers will extract data into a data extraction sheet and assess 25% of the studies, with disagreements resolved by discussion, deferring to a third reviewer where consensus cannot be achieved. The remaining 75% of studies will be extracted by one reviewer. Extraction will include key descriptive information regarding each study (e.g., population, methods, outcomes). The piloted data extraction sheet is provided within Supplementary Material 3.

### Quality Assessment and Strength of Evidence

Risk of bias and quality assessment will be assessed at the individual study level using the relevant Joanna Briggs Institute critical appraisal tool (Joanna Briggs Institute, 2023) and at the outcome level using the GRADE tool (Grading of Recommendations, Assessment, Development, and Evaluations) (Guyatt et al. 2008). Two independent reviewers will assess 25% of studies, with disagreements resolved by discussion, deferring to a third reviewer where consensus cannot be achieved. The remaining 75% of studies will be assessed by one reviewer.

## Data Synthesis

A narrative synthesis will provide an initial descriptive overview of the search results and included studies before meta-analysis and/or Synthesis without Meta-Analysis (SWiM, Campbell et al., 2020). Where possible, findings will be presented both overall and grouped by country, allowing data to be presented in the context of different healthcare models.

### Meta-Analysis

Where possible, data will be pooled in a meta-analysis. It is anticipated that outcomes are likely to be quantified in terms of mean visits per time period (e.g., mean annual hospitalizations), odds or rate ratios (e.g., the incident rate ratio for total annual prescriptions between people with fibromyalgia alone, compared to those with comorbid depression), and associated costs per annum for either a fibromyalgia population only or with a comparator. In this instance, a meta-analysis of continuous outcomes will be conducted using mean difference (Deeks, Higgins & Altman, 2023). Prevalence, count and rate data (e.g., proportion of analgesic prescriptions between people with fibromyalgia and controls) may also be collected, in which instance the appropriate analyses will be chosen based on the Cochrane Handbook (Deeks, Higgins & Altman, 2023).

A random or fixed effects model will be selected based on study heterogeneity. Heterogeneity will be explored using subgroup analyses, and if data allow meta-regression, giving specific reference to geography and sample size.

Individual study and combined effect estimates will be presented using Forest Plots, along with the reporting of confidence intervals and *p*-values.

### Publication Bias

Where possible, publication bias will be assessed using plots and appropriate statistical procedures (e.g. Egger’s test). If there is evidence of potential publication bias, approaches such as the trim-and-fill method (Duval & Tweedie, 2000) will be used to identify bias and adjust data.

### Subgroup and Sensitivity Analysis

Subgroup analyses may be conducted to explore the effect of different healthcare models, patient co-morbidities or characteristics, alongside variation in healthcare use over time. Where possible, a sensitivity analysis will explore the impact of low-quality studies and any uncertain/missing data on the combined effect estimate.

### SWiM

For studies not suitable for inclusion in a meta-analysis (e.g., lacking relevant data), SWiM guidelines (Campbell et al., 2020) will be adhered to. Results may be synthesized through vote counting based on direction of effect. Depending on data availability, vote counting for within fibromyalgia comparisons may be stratified by patient characteristics (e.g., age or comorbidity), time-periods, type of service, country or stage of fibromyalgia (e.g., pre- vs post-diagnosis).

Tabular summaries will report summary statistics (e.g., mean, standard deviation, *p*-values). Where a key summary statistic is not provided these will be calculated where possible using Stata (StataCorp, 2023). Data may also be presented using an appropriate visualization (e.g., Forest or Albatross Plots) (Campbell et al., 2020).

### Patient and Public Involvement and Engagement (PPIE)

A PPIE group will be convened comprising people with lived experience of fibromyalgia to inform the conduct of the review and interpretation of its findings.

## Discussion

Fibromyalgia is a common long-term condition that is characterized by widespread pain and multiple somatic symptoms, requires early diagnosis alongside a focus on holistic non-pharmacological care, and is considered in many countries to be best managed predominantly in primary care and the community (National Institute for Health and Care Excellence, 2021; McConnell et al., 2023). Despite this, evidence indicates that people with fibromyalgia wait many years to receive a diagnosis, often receive long-term analgesics, and are commonly seen in secondary care. Our review will help resolve this gap between recommendations and practice by synthesising global data on healthcare use in people with fibromyalgia and understanding how it is changing over time. Through evaluating how healthcare use varies between geographical regions, it can be used to inform health service planning and future guideline/policy development (although any recommendations for policy and practice would need to consider implementation factors alongside theory and context for any evidence-to-practice gaps). Additionally, by exploring whether groups of people with fibromyalgia exist with particularly high levels of healthcare use, it could lead to the identification of sub-populations most likely to benefit from tailored interventions and care.

This review’s strengths will include: 1) its adherence to PRISMA guidelines; 2) registration of a protocol, containing a pre-defined synthesis strategy and methods; 3) development of a search strategy with clinical and academic input; and 4) adherence to Cochrane recommendations. Its potential limitations include: 1) its intention to synthesize global evidence, but the exclusion of articles written in non-English languages for which translation cannot be attained (although we anticipate that, between the use of translation software and native speakers, this will be minimal); 2) anticipated heterogeneity across study outcomes, potentially limiting the ability to conduct a meta-analysis; 3) possible unsuitability of synthesising evidence from a range of healthcare systems, which have different models of care for people with fibromyalgia, and 4) whilst a PPIE group will be consulted on the conduct and interpretation of the review, patients were not involved in the design. This may bias the relevance of the outcome measures to clinicians and overlook potentially important outcomes to patients. Whilst restricting the design of included studies to EHR, health insurance, and registry datasets could be considered a potential limitation, this design allows the integration and analysis of large volumes of diverse and representative datasets, avoids including large numbers of small observational studies (e.g., case series) that would add little to the review findings, and avoids including trials, which are likely to recruit people who are not representative of the full spectrum of fibromyalgia and in whom healthcare use is not generalizable. Although we will not consider qualitative studies within our literature search (as they are not well placed to answer the review questions) we will consider relevant qualitative studies when interpreting our systematic review’s findings, to provide insight into why any patterns and trends in healthcare use are seen.

Through the synthesis and analysis of global data on healthcare use and its variation in people with fibromyalgia, it is anticipated that this review’s findings may be used to bridge the gap between policy and practice at an international level, help support integration of healthcare services and provide vital insights to facilitate the increasing transition towards managing fibromyalgia within primary care.

## Supporting information

Byrne et al. supplementary material 1 Byrne et al. supplementary material

Byrne et al. supplementary material 2 Byrne et al. supplementary material

Byrne et al. supplementary material 3 Byrne et al. supplementary material

## Supplementary material

The supplementary material for this article can be found at https://doi.org/10.1017/S1463423625000362
