# Supplementary material for: Healthcare use and its variation in people with fibromyalgia: a systematic review protocol
Source: Prim Health Care Res Dev. 2025 May 7;26:e42. doi: 10.1017/S1463423625000362 (PMC12099268; doi:10.1017/S1463423625000362)
Supplement: Byrne et al. supplementary material 2 — Byrne et al. supplementary material [file S1463423625000362sup002.pdf]

Manuscript: Healthcare Use and its Variation in People with Fibromyalgia: A Systematic Review Protocol
Journal: Primary Health Care Research and Development
Authors: Ailish Katherine Byrne, Helen Twohig, Sara Muller and Ian C Scott
Corresponding Author: Ailish Katherine Byrne, Keele University, a.k.byrne@keele.ac.uk

Supplementary Material 2: Search strategy

MEDLINE	
	**Term**
1.	(MM "Fibromyalgia")
2.	AB fibromyalgia OR TI fibromyalgia OR SU fibromyalgia
3.	1 OR 2
4.	AB healthcare OR TI healthcare OR SU healthcare
5.	AB health care OR TI health care OR SU health care
6.	AB health service OR TI health service OR SU health service
7.	AB inpatient OR TI inpatient OR SU inpatient
8.	AB outpatient OR TI outpatient OR SU outpatient
9.	AB ("accident and emergency") OR TI ("accident and emergency") OR SU ("accident and emergency")
10.	AB "emergency department" OR TI "emergency department" OR SU "emergency department"
11.	AB primary care OR TI primary care OR SU primary care
12.	AB secondary care OR TI secondary care OR SU secondary care
13.	(MH "Health Services+")
14.	(MH "Inpatients") OR (MH "Outpatients") OR (MH "Patients")
15.	(MH "Hospital+")
16.	(MH "Primary Health Care+") OR (MH "Physicians, Primary Care")
17.	(MH "Tertiary Healthcare") OR (MH "Secondary Care")
18.	(MH "Prescription Drugs")
19.	(MH "Referral and Consultation+")
20.	AB tertiary care OR TI tertiary care OR SU tertiary care *
21.	AB rheum* OR TI rheum* OR SU rheum*
22.	AB prescr* OR TI prescr* OR SU prescr*
23.	AB opioid OR TI opioid OR SU opioid
24.	AB antidepressant OR TI antidepressant OR SU antidepressant
25.	AB analges* OR TI analges* OR SU analges*
26.	AB medication OR TI medication OR SU medication
27.	AB consult* OR TI consult* OR SU consult*
28.	4 – 27 OR
29.	AB electronic health record OR TI electronic health record OR SU electronic health record
30.	AB (register or registry or registries) OR TI (register or registry or registries) OR SU (register or registry or registries)
31.	AB database OR TI database OR SU database
32.	AB insurance OR TI insurance OR SU insurance
33.	AB routine data OR TI routine data OR SU routine data
34.	AB medical record OR TI medical record OR SU medical record
35.	AB CPRD OR TI CPRD OR SU CPRD
36.	AB QResearch OR TI QResearch OR SU QResearch

37.	AB Health Improvement Network OR TI Health Improvement Network OR SU Health Improvement Network
38.	AB Medical Information Mart for Intensive Care OR TI Medical Information Mart for Intensive Care OR SU Medical Information Mart for Intensive Care
39.	AB Medicare OR TI Medicare OR SU Medicare
40.	AB Medicaid OR TI Medicaid OR SU Medicaid
41.	AB health maintenance organization OR TI health maintenance organization OR SU health maintenance organization
42.	29 – 41 OR
43.	28 AND 42
44.	3 AND 43

**Web of Science**	
	**Term**
1.	TS=(fibromyalgia)
2.	((AB=("fibromyalgia")) OR TI=("fibromyalgia"))
3.	#1 OR #2
4.	((AB=("healthcare")) OR TI=("healthcare"))
5.	((AB=("health care")) OR TI=("health care"))
6.	((AB=("health service")) OR TI=("health service"))
7.	((AB=("inpatient")) OR TI=("inpatient"))
8.	((AB=("outpatient")) OR TI=("outpatient"))
9.	((AB=("accident and emergency")) OR TI=("accident and emergency"))
10.	((AB=("emergency department")) OR TI=("emergency department"))
11.	((AB=("primary care")) OR TI=("primary care"))
12.	((AB=("secondary care")) OR TI=("secondary care"))
13.	TS=(health services)
14.	TS=("Inpatients") OR TS=("Outpatients") OR TS=("Patients")
15.	TS=("Hospital")
16.	TS=("Primary health care+") OR TS=("Physicians, Primary care")
17.	TS=("Secondary healthcare+") OR TS=("Tertiary Healthcare")
18.	TS=("Prescription Drugs")
19.	TS=("Referral and consultation")
20.	((AB=("tertiary care")) OR TI=("tertiary care"))
21.	((AB=("rheum*")) OR TI=("rheum*"))
22.	((AB=("prescr*")) OR TI=("prescr*"))
23.	((AB=("opioid")) OR TI=("opioid"))
24.	((AB=("antidepressant")) OR TI=("antidepressant"))
25.	((AB=("analges*")) OR TI=("analges*"))
26.	((AB=("medication")) OR TI=("medication"))
27.	((AB=("consult*")) OR TI=("consult*"))
28.	#4 – #27 OR
29.	((AB=("electronic health record")) OR TI=("electronic health record"))
30.	((AB=("regist*")) OR TI=("regist*"))

31.	((AB=("database")) OR TI=("database"))
32.	((AB=("insurance")) OR TI=("insurance"))
33.	((AB=("routine data")) OR TI=("routine data"))
34.	((AB=("medical record")) OR TI=("medical record"))
35.	((AB=("CPRD")) OR TI=("CPRD"))
36.	((AB=("Qresearch")) OR TI=("Qresearch"))
37.	((AB=("Health Improvement Network")) OR TI=("Health Improvement Network"))
38.	((AB=("Medical Information Mart for Intensive Care")) OR TI=("Medical Information Mart for Intensive Care"))
39.	((AB=("Medicare")) OR TI=("Medicare"))
40.	((AB=("Medicaid")) OR TI=("Medicaid"))
41.	((AB=("health maintenance organization")) OR TI=("health maintenance organization"))
42.	#29 – #41 OR
43.	#42 and #28
44.	#3 AND #43

**CINHAL**	
	**Term**
1.	MM Fibromyalgia
2.	AB fibromyalgia OR TI fibromyalgia OR SU fibromyalgia
3.	1 OR 2
4.	AB healthcare OR TI healthcare OR SU healthcare
5.	AB health care OR TI health care OR SU health care
6.	AB health service OR TI health service OR SU health service
7.	AB inpatient OR TI inpatient OR SU inpatient
8.	AB outpatient OR TI outpatient OR SU outpatient
9.	AB ("accident and emergency") OR TI ("accident and emergency") OR SU ("accident and emergency")
10.	AB "emergency department" OR TI "emergency department" OR SU "emergency department"
11.	AB primary care OR TI primary care OR SU primary care
12.	AB secondary care OR TI secondary care OR SU secondary care
13.	(MH "Health Services+")
14.	(MH "Inpatients") OR (MH "Outpatients") OR (MH "Patients")
15.	(MH "Hospital+")
16.	(MH "Primary Health Care+") OR (MH "Physicians, Primary Care")
17.	(MH "Tertiary Healthcare") OR (MH "Secondary Care")
18.	(MH "Prescription Drugs")
19.	(MH "Referral and Consultation+")
20.	AB tertiary care OR TI tertiary care OR SU tertiary care *
21.	AB rheum* OR TI rheum* OR SU rheum*
22.	AB prescr* OR TI prescr* OR SU prescr*
23.	AB opioid OR TI opioid OR SU opioid
24.	AB antidepressant OR TI antidepressant OR SU antidepressant

25.	AB analges* OR TI analges* OR SU analges*
26.	AB medication OR TI medication OR SU medication
27.	AB consult* OR TI consult* OR SU consult*
28.	4 – 27 OR
29.	AB electronic health record OR TI electronic health record OR SU electronic health record
30.	AB (register or registry or registries) OR TI (register or registry or registries) OR SU (register or registry or registries)
31.	AB database OR TI database OR SU database
32.	AB insurance OR TI insurance OR SU insurance
33.	AB routine data OR TI routine data OR SU routine data
34.	AB medical record OR TI medical record OR SU medical record
35.	AB CPRD OR TI CPRD OR SU CPRD
36.	AB QResearch OR TI QResearch OR SU QResearch
37.	AB Health Improvement Network OR TI Health Improvement Network OR SU Health Improvement Network
38.	AB Medical Information Mart for Intensive Care OR TI Medical Information Mart for Intensive Care OR SU Medical Information Mart for Intensive Care
39.	AB Medicare OR TI Medicare OR SU Medicare
40.	AB Medicaid OR TI Medicaid OR SU Medicaid
41.	AB health maintenance organization OR TI health maintenance organization OR SU health maintenance organization
42.	29 – 41 OR
43.	28 AND 42
44.	3 AND 43

**Embase**	
	**Term**
1.	Exp *fibromyalgia/
2.	fibromyalgia.ti. or fibromyalgia.ab. or fibromyalgia.sh.
3.	1 OR 2
4.	healthcare.ti. or healthcare.ab. or healthcare.sh.
5.	health care.ti. or health care.ab. or health care.sh.
6.	health service.ti. or health service.ab. or health service.sh.
7.	inpatient.ab. or inpatient.ti. or inpatient.sh.
8.	outpatient.ti. or outpatient.ab. or outpatient.sh.
9.	(accident and emergency).ti. or (accident and emergency).ab. or (accident and emergency).sh.
10.	emergency department.ti. or emergency department.ab. or emergency department.sh.
11.	primary care.ti. or primary care.ab. or primary care.sh.
12.	secondary care.ti. or secondary care.ab. or secondary care.sh.
13.	exp *health services/
14.	exp *inpatient/ or exp *outpatient/ or exp *patient/
15.	exp *hospital/

16.	exp *Primary Health Care/ or exp *Physicians, Primary Care/
17.	exp *Tertiary Healthcare/ or exp *Secondary Care/
18.	exp *prescription/ or *drug/
19.	exp *referral/ or exp *consultation/
20.	tertiary care.ti. or tertiary care.ab. or tertiary care.sh.
21.	rheumatology.ti. or rheumatology.ab. or rheumatology.sh.
22.	prescription.ti. or prescription.ab. or prescription.sh.
23.	opioid.ti. or opioid.ab. or opioid.sh.
24.	antidepressant.ti. or antidepressant.ab. or antidepressant.sh.
25.	analgesic.ti. or analgesic.ab. or analgesic.sh.
26.	medication.ti. or medication.ab. or medication.sh.
27.	consultation.ti. or consultation.ab. or consultation.sh.
28.	4 – 27 OR
29.	electronic health record.ti. or electronic health record.ab. or electronic health record.sh.
30.	registry.ti. or registry.ab. or registry.sh.
31.	database.ti. or database.ab. or database.sh.
32.	insurance.ti. or insurance.ab. or insurance.sh.
33.	routine data.ti. or routine data.ab. or routine data.sh.
34.	medical record.ti. or medical record.ab. or medical record.sh.
35.	CPRD.ti. or CPRD.ab. or CPRD.sh.
36.	Qresearch.ti. or Qresearch.ab. or Qresearch.sh.
37.	Health Improvement Network.ti. or Health Improvement Network.ab. or Health Improvement Network.sh.
38.	Medical Information Mart for Intensive Care.ti. or Medical Information Mart for Intensive Care.ab. or Medical Information Mart for Intensive Care.sh.
39.	Medicare.ti. or Medicare.ab. or Medicare.sh.
40.	Medicaid.ti. or Medicaid.ab. or Medicaid.sh.
41.	health maintenance organization.ti. or health maintenance organization.ab. or health maintenance organization.sh.
42.	29 – 41 OR
43.	28 AND 42
44.	3 AND 43
