# Supplementary material for: Healthcare use and its variation in people with fibromyalgia: a systematic review protocol
Source: Prim Health Care Res Dev. 2025 May 7;26:e42. doi: 10.1017/S1463423625000362 (PMC12099268; doi:10.1017/S1463423625000362)
Supplement: Byrne et al. supplementary material 3 — Byrne et al. supplementary material [file S1463423625000362sup003.pdf]

Overview of included studies																			
Author	Year	Country	Population	FM sample size	Control sample size	Years data collected from	EHR used	Overall FM age	Overall control age	FM number (%) female	FM number (%) male	Control number (%) female	Control number (%) male	FM ethnicity (number(%))	Control ethnicity (number(%))	FM BMI	Control BMI	FM SES (e.g.: low, medium, high)	Control SES (e.g.: low, medium, high)

FM smokers (N, %)	Control smokers (N, %)	Healthcare services assessed	Outcome measures	Funder/ Sponsor	Other notes

Study overview				GP visits							
Author	Year	What comparison is being made?	Time frame	Mean (SD)	**Odds or rate ratio (95% CIs)	Confidence intervals	Proportion N (%)	p.value	Other relevant statistic	Mean (SD)	**Odds or rate ratio (95% CIs)

Referrals				Prescription (overall)						Prescription (analgesics)						Prescriptions (a		
Confidence intervals	Proportion N (%)	p.value	Other relevant statistic	Mean (SD)	**Odds or rate ratio (95% CIs)	Confidence intervals	Proportion N (%)	p.value	Other relevant statistic	Mean (SD)	**Odds or rate ratio (95% CIs)	Confidence intervals	Proportion N (%)	p.value	Other relevant statistic	Mean (SD)	**Odds or rate ratio (95% CIs)	Confidence intervals

nti-depressant)			Prescriptions (other relevant drugs)						Outpatient visits						Inpatient visits/Hospitalisa			
Proportion N (%)	p.value	Other relevant statistic	Mean (SD)	**Odds or rate ratio (95% CIs)	Confidence intervals	Proportion N (%)	p.value	Other relevant statistic	Mean (SD)	**Odds or rate ratio (95% CIs)	Confidence intervals	Proportion N (%)	p.value	Other relevant statistic	Mean (SD)	**Odds or rate ratio (95% CIs)	Confidenc e intervals	Proportion N (%)

ation		A&E/ED visits						Pain clinic						Rheumatology clinic visits					
p.value	Other relevant statistic	Mean (SD)	**Odds or rate ratio (95% CIs)	Confidence intervals	Proportion N (%)	p.value	Other relevant statistic	Mean (SD)	**Odds or rate ratio (95% CIs)	Confidence intervals	Proportion N (%)	p.value	Other relevant statistic	Mean (SD)	**Odds or rate ratio (95% CIs)	Confidence intervals	Proportion N (%)	p.value	Other relevant statistic

Investigations (laboratory tests, imaging etc.)						Overall healthcare resource use						Comorbidity	Study information			
Mean (SD)	**Odds or rate ratio (95% CIs)	Confidence intervals	Proportion N (%)	p.value	Other relevant statistic	Mean (SD)	**Odds or rate ratio (95% CIs)	Confidence intervals	Proportion N (%)	p.value	Other relevant statistic		Author	Year	What comparison is being made?	Time frame

Rates of comorbidity at baseline per annum (specify if other)				Overall healthcare resource use (e.g.: count of visits) for subgroup with comorbidity vs without per					Overall medication use/prescription count for subgroup with comorbidity vs without per annum					Overall healthcare cost for subgroup with comorbidity vs without per annum (specify if other)				
Percentage FM patients with comorbidi	Percentage control patients with comorbidi	**Odds or rate ratio (95% CIs) (specify)	p-value	Mean (SD)	**Odds or rate ratio (95% CIs) (specify)	Proportion as N(%)	p.value	Other relevant statistic	Mean (SD)	**Odds or rate ratio (95% CIs) (specify)	Proportion as N(%)	p.value	Other relevant statistic	Mean (SD)	**Odds or rate ratio (95% CIs) (specify)	Proportion as N(%)	p.value	Other relevant statistic

					Overall healthcare resource use per annum						Overall medication use per annum						
Characteristic	Author	Year	What is the comparison being made?	Time frame of outcome	Mean (SD)	Odds or rate ratio (specify)	95% confidence intervals	Proportion N(%)	p.value	Other relevant statistic	Mean (SD)	Odds or rate ratio (specify)	95% confidence intervals	Proportion N(%)	p.value	Other relevant statistic	Mean (SD)

Overall healthcare cost per annum					Overall health service use/medical cost			Prescriptions (antidepressant cost)			Prescriptions (other relevant drugs) cost			GP/Family Physician visit cost			Outpa
Odds or rate ratio (specify)	95% confidence intervals	Proportion N(%)	p.value	Other relevant statistic	Mean (SD)	p.value	Other relevant statistic	Mean (SD)	p.value	Other relevant statistic	Mean (SD)	p.value	Other relevant statistic	Mean (SD)	p.value	Other relevant statistic	Mean (SD)

atient visit costs		Inpatient visits/Hospitalisation cost			A&E/ED visits cost			Pain clinic visit cost			Rheumatology clinic visit cost			Investigations (e.g: tests & imaging) cost			Other healthcare vi	
p.value	Other relevant statistic	Mean (SD)	p.value	Other relevant statistic	Mean (SD)	p.value	Other relevant statistic	Mean (SD)	p.value	Other relevant statistic	Mean (SD)	p.value	Other relevant statistic	Mean (SD)	p.value	Other relevant statistic	Mean (SD)	p.value

sit costs
Other relevant statistic
